# Repetitive Gamma-tACS Improves the Reaction Times of Healthy Young Adults in a Visuospatial Working Memory Task: A Randomized Study

**DOI:** 10.3390/brainsci15040343

**Published:** 2025-03-27

**Authors:** Miriam Rosato, Marco Sala, Ambra Coccaro, Simone Cutini, Mario Liotti

**Affiliations:** 1 Department of Developmental and Social Psychology, University of Padova, Via Venezia 8, 35131 Padova, Italy; miriam.rosato@unipd.it (M.R.); marco.sala.2@studenti.unipd.it (M.S.); ambra.coccaro@colorado.edu (A.C.); simone.cutini@unipd.it (S.C.); 2 Padova Neuroscience Center, University of Padova, Via Orus 2/B, 35129 Padova, Italy; 3 Institute of Cognitive Sciences, University of Colorado, Boulder, CO 80309, USA; 4 Department of Psychology, Simon Fraser University, Burnaby, BC V5A 1S6, Canada

**Keywords:** tACS, neuromodulation, visuospatial working memory, gamma-band

## Abstract

**Objective**: The aims of the study were to test the short-term and long-term efficacy of repetitive γ-tACS over the left DLPFC to improve visuospatial working memory performance in the spatial capacity delayed response task (SCDRT). **Methods**: In a single blind placebo-controlled study, 35 healthy young adults were randomly assigned to three sessions of either active γ-tACS (n = 18) or passive sham γ-tACS (n = 17) The design allowed us to evaluate the influence of the stimulation protocol (active vs. sham), the stimulation session number (day 1 to 3), the session block (before stimulation, during stimulation and after stimulation) and the VSWM retention load (1, 3, 5 or 7 stimuli) on the response speed and accuracy. **Results**: Active γ-tACS selectively improved VSWM performance on day 2 and 3, and the effect was greater following stimulation rather than during stimulation. Significant effects were seen concerning response speed but not accuracy. The VSWM performance gains of the active γ-tACS were no longer present in the long-term at a follow-up session after two weeks. **Conclusions**: The present study provides novel evidence for a selective improvement in VSWM performance with three repeated sessions of γ-tACS in young adults through the entrainment of gamma rhythms in the left DLPFC.

## 1. Introduction

Transcranial alternating current stimulation (tACS) is a non-invasive electric stimulation technique believed to affect brain functioning by the neuromodulation of intrinsic rhythmic activity [1,2]. TACS-induced neural entrainment would promote the synchronization of phasic neural oscillations at specific frequencies, with effects on cognitive performance and brain function [3]. Recent times have indeed seen a growing interest in employing tACS in the field of cognitive neuroscience, both in healthy and neuropsychiatric groups. In particular, a growing number of studies in recent years have employed tACS both to demonstrate the causal relationship between neural oscillations and working memory (WM) performance, and to improve WM functioning in a clinical setting and in the elderly population with WM deficits [4,5].

Visuospatial working memory (VSWM) is defined as the capacity to maintain and manipulate visuospatial information across a brief delay. Research on the cognitive neuroscience of VSWM has revealed a network of brain regions involved in VSWM maintenance and manipulation, including the dorsolateral prefrontal cortex (DLPFC), parietal cortex, anterior cingulate cortex and frontal eye fields. Particularly relevant are studies evaluating the influence on the DLPFC activity of the VSWM retention load by employing the spatial capacity delayed response task (SCDRT; [6,7]).

### 1.1. WM and Neural Oscillations

Oscillatory activity is thought to crucially support the maintenance of information in the WM, with distinct functional roles for theta (θ: 4–7 Hz), alpha (α: 8–12 Hz) and gamma (γ: 30–80 Hz) frequencies; specifically, γ-band oscillations would be involved in the maintenance of WM information, α-band activity would reflect the active inhibition of task-irrelevant information, whereas θ-band oscillations would underlie the organization of sequentially ordered WM items [8]. It has also been proposed that the coordination and maintenance of representational objects in the visual WM requires intra-areal synchrony between α-, β- and γ-frequency bands in the fronto-parietal areas and in the visual areas [9].

With regard to VSWM, studies have reported associations between oscillatory patterns and the spatial short-term memory, in particular, mental rotation, especially in the γ-band [10,11], but also in the beta-band (β: 13–29 Hz) [12]. Of relevance was a review concluding that, in working memory tasks, γ- and β-bands address different processing stages. The γ-frequency would be prevalent during the presentation of the item to be held in memory, while the β-band would be prominent during the maintenance period [13].

In fact, it has been proposed that the bottom-up or perceptually driven processes are mediated by the local γ-frequency, whereas top-down processes would involve long-distance oscillations in the β-, α- and θ-bands [14]. The γ-frequency band has also been linked to perceptual binding, that is, the process whereby the sensory stimuli are combined together in order to create a meaningful and unitary percept. Finally, a recent high-density resting state EEG study by our research group found that functional connectivity in the γ-band in nodes of the left DAN (superior frontal–intraparietal network) predicted the performance of typically developing children and adolescents on the Rey–Osterrieth complex figure test (ROCFT), which is used to assess visuospatial memory and visuoconstructive abilities [15].

### 1.2. Neuromodulation and WM

Several studies employing transcranial electric or magnetic stimulation have investigated the effects on brain regions specifically associated with WM. In a study applying anodal transcranial direct current stimulation (tDCS) over the left DLPFC either during (*online*) or before (*offline*) the execution of an n-back task, a significant improvement in digit span performance was found, but only for the *online* stimulation [16].

A meta-analysis exploring the effects of tDCS and repetitive transcranial magnetic stimulation (rTMS) on DLPFC in n-back tasks in healthy and neuropsychiatric cohorts found an overall improvement in reaction times (RTs) for tDCS and rTMS, and an improvement in accuracy for rTMS [17]. A second meta-analysis compared the effects of tDCS, tACS and rTMS on mental rotation ability as a component of VSWM. The results showed a beneficial effect of anodal tDCS and tACS on mental rotation performance, with no effect of cathodal tDCS [18]. A third meta-analysis assessed the effects of stimulation with anodal tDCS in healthy and neuropsychiatric populations, measuring RTs and accuracy in WM tasks (n-back, Sternberg task and digit span). The results revealed significant improvements in WM performance (RTs and accuracy) in healthy cohorts, but only for the *offline* stimulation and not for the online treatment [19]. Similarly, a further recent meta-analysis indicated greater improvements in cognitive function following tACS (*offline* effects) than during tACS stimulation (*online* effects) [20].

Table 1 below summarizes the methods and results of published papers reporting the effects of tACS on WM in healthy adults. A first study reported that γ-tACS (but not tDCS) over the left frontal cortex improved WM performance in the n-back task, with a significant effect for the 3-back relative to the 2-back condition (d-prime scores but not RTs) [21]. However, other studies employing γ-tACS over the bilateral frontal cortex found no effects on WM, as assessed by the n-back task [22] or a visual WM task (the Luck and Vogel change detection task) [23]. Another study compared the effects of γ-tACS or θ-tACS over the bilateral frontal, bilateral parietal, left fronto-parietal or right fronto-parietal cortex on WM performance in n-back tasks and change detection tasks. No behavioral effects obtained significance, while some EEG changes were found for θ-tACS following stimulation [24]. According to the authors, a single stimulation session may have not been sufficient to yield WM gains; and future studies may be necessary to evaluate the effects of repeated stimulation sessions [22,23,24].

Technical advancements of the tACS equipment and procedure may improve the ability to produce and detect cognitive changes. In fact, two recent studies employed high definition (HD) tACS—allowing for a more focal target of stimulation—and the combined γ-tACS and θ-tACS stimulation of the left frontal cortex to evaluate the effects of a single session of stimulation on WM performance in various tasks (visuospatial WM task, Sternberg task, Flanker task, Digit Symbol Substitution Task [DSST], Wisconsin Card Sorting Task) [25,26]. For the Sternberg task, the young adults’ accuracy increased for the higher load [26], whereas for elder adults, the RT performance improved (but accuracy decreased) for both the lower and higher load [25]. No effect was found for the visuospatial WM task [25,26].

As suggested earlier, the efficacy of neurostimulation techniques in producing cognitive gains and broadening their clinical applications may be increased by the use of repeated stimulation sessions. Previous research suggests that increasing the number of tACS sessions, e.g., one session per day for five consecutive days per week, can significantly improve responses to stimulation. This approach may lead to cumulative and long-lasting effects through the mechanisms that promote neuroplasticity [27,28]. To our knowledge, a single recent study tested the efficacy of repetitive HD-tACS stimulation (four sessions on consecutive days) to improve performance in a verbal memory task in elderly healthy participants. A significant effect on WM (immediate recall) was present on days 3 and 4 of active θ-tACS over the left parietal cortex, suggesting that repeated sessions are indeed required to obtain a performance improvement in WM tasks. Notably, the WM gains were still present at a follow-up session after one month [29].

**Table 1:** Summary of studies investigating the effects of tACS on WM in healthy adults (ACC: accuracy, RTs: reaction times); target locations in 10–20 coordinate system (e.g., F3 or AF3); DLPFC: dorsolateral prefrontal cortex; IPL: inferior parietal lobule; DSST: Digit Symbol Substitution Task; WCST: Wisconsin Card Sorting Test.

**Palm et al., 2014** [22]	
Band frequency (tACS):	Gamma (40 Hz)-tACS
Study design:	Between-subjects (active/sham)
Number of active session(s):	1
Region(s) of stimulation:	Bilateral frontal cortex (F3 and F4)
Participant characteristics:	Healthy volunteers and major depression patients
Task(s)—online/offline:	n-Back task (2-back and 3-back)—online
Significant results:	No effects independent of retention load
DOI:	10.1016/j.neucli.2022.03.002
**Hoy et al., 2015** [21]	
Band frequency (tACS):	3 sessions: gamma(40 Hz)-tACS + tDCS + sham
Study design:	Within subjects (active/tDCS/sham)
Number of active session(s):	2 (one tACS + one tDCS)
Region(s) of stimulation:	Left frontal cortex (F3)
Participant characteristics:	Young adults
Task(s)—online/offline:	2-back (online and offline) 3-back (offline)
Significant results:	d-prime: For γ-tACS only, larger improvement in 3-back than 2-back. RT: no effects.
DOI:	10.1016/j.bandc.2015.11.002
**Kvašňák et al., 2018** [23]	
Band frequency (tACS):	Gamma (40 Hz)-tACS
Study design:	Between-subjects (active/sham)
Number of active session(s):	1
Region(s) of stimulation:	Bilateral frontal cortex (F3 and F4)
Participant characteristics:	Young adults
Task(s)—online/offline:	Visual WM task (Luck and Vogel paradigm)—offline
Significant results:	None
DOI:	10.3390/bs13010039
**Pahor et al., 2018** [24]	
Band frequency (tACS):	Theta-tACS or gamma-tACS
Study design:	Within-subjects (theta/gamma/sham) Between-subjects (regions of stimulation)
Number of active session(s):	2 (one theta-tACS + one gamma-tACS)
Region(s) of stimulation:	4 groups: bilateral parietal (P3-P4), left fronto-parietal (F3-P3), right fronto-parietal (F4-P4), bilateral frontal (F3-F4)
Participant characteristics:	Young adults
Task(s)—online/offline:	Change detection tasks (figural and verbal)—offline. N-back tasks (figural and verbal variants of 2- and 3-back tests)—offline
Significant results:	No behavioral effects. Offline: active theta-tACS increased P3 component during n-back tasks in the bilateral parietal and right fronto-parietal protocols.
DOI:	10.3389/fnhum.2017.00651
**Grover et al., 2022** [29]	
Band frequency (tACS):	Gamma (60 Hz)-HD-tACS or theta (4 Hz)-HD-tACS
Study design:	Between-subjects (active/sham)
Number of active session(s):	4 (day 1, 2, 3 and 4)
Region(s) of stimulation:	DLPFC (AF3) or IPL (CP5)
Participant characteristics:	Old adults (69–88 ys old)
Task(s)—online/offline:	Free recall task (online for stimulation days, offline at baseline pre-stimulation and one month follow-up)
Significant results:	Theta-frequency in IPL improved WM on day 3 and 4 and 1 month after intervention. Gamma-frequency in DLPFC improved LTM on days 2–4 and 1 month after intervention.
DOI:	10.1038/s41593-022-01132-3
**Abubaker et al., 2024** [25]	
Band frequency (tACS):	Theta (6 Hz)–gamma (80 Hz) peak coupled HD-tACS
Study design:	Within subjects (active/sham)
Number of active session(s):	1
Region(s) of stimulation:	Left frontal cortex (F3)
Participant characteristics:	Old adults
Task(s)—online/offline:	Visuospatial WM task, Sternberg task, Flanker task, DSST, WCST (online)
Significant results:	Decrease in ACC and RTs on the 10- and 14-item Sternberg tasks. Increase in RTs on the DSST.
DOI:	10.1186/s13041-024-01149-8
**Al Qasem et al., 2024** [26]	
Band frequency (tACS):	Theta (6 Hz)–gamma (80 Hz) peak coupled HD-tACS
Study design:	Within subjects (active/sham)
Number of active session(s):	1
Region(s) of stimulation:	Left frontal cortex (F3)
Participant characteristics:	Young adults
Task(s)—online/offline:	Visuospatial WM task, Sternberg task, Flanker task, DSST, WCST (online)
Significant results:	Increase in ACC only on the 14-item Sternberg task.
DOI:	10.1186/s13041-024-01142-1

### 1.3. Study Rationale and Aims

**Figure 1:**
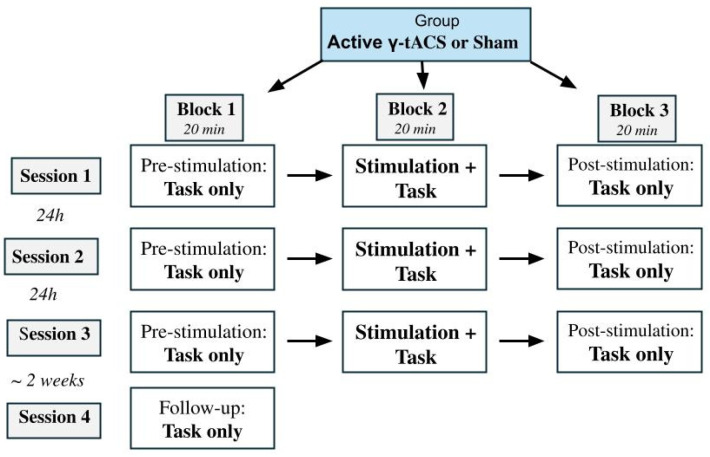
Illustration of the study design, showing Groups, Sessions and Blocks.

To our knowledge, no studies have been published evaluating the effects of repetitive tACS (r-tACS) on VSWM. Based on evidence that the γ-frequency band is particularly implicated in various aspects of VSWM, which includes the short-term maintenance of visual and visuospatial information [8,9], given the role of DLPFC as the main hub in the cortical network involved in VSWM maintenance and manipulation [6,7] and the resting EEG evidence that γ-band connectivity in the left DAN predicts visuospatial performance in the ROCFT [15], we selected the γ-band (40 Hz) as the tACS stimulation frequency and the left DLPFC as the target region of interest (ROI) for stimulation. We also opted for employing the SCDRT as a well-validated task to study the VSWM performance and the related DLPFC activity as a function of retention load [6,7], given the evidence that γ-tACS improves WM performance at higher loads in an n-back task [21]. Based on recent proof that r-tACS boosted verbal WM performance (immediate recall) starting from stimulation day 3, with long-term gains of up to a month [29], we opted to evaluate the short- and long-term efficacy of a three-day active r-tACS intervention protocol. Given the inconsistencies in the efficacy of online vs. offline stimulation on cognitive performance, we decided to test both the online and offline effects within each session. Please refer to Figure 1 as an illustration of the study design.

The main aim of the present study was therefore to evaluate the short-term efficacy of repeated γ-tACS over the left DLPFC to improve the VSWM performance in healthy young adults, as a function of stimulation type (active vs. passive sham), stimulation session number (day 1 to day 3), session block (before, during and after tACS) and WM retention load (1 to 7 stimuli). The second aim of this study was to evaluate the possible long-term effects of active repetitive γ-tACS by evaluating VSWM gains with a follow-up session after two weeks.

### 1.4. Hypotheses

The first experimental prediction was that the active r-tACS intervention would significantly improve VSWM performance (accuracy and/or response speed) relative to the passive sham tACS treatment by a progressive boosting of VSWM over the three stimulation sessions, as reported by a recent verbal WM study [29].

The second experimental prediction was that the active r-tACS-induced VSWM performance improvement relative to the sham intervention would be significantly greater in the post-stimulation block (*offline* effect) relative to the stimulation block (*online* effect), consistent with recent literature [19,20].

The third experimental hypothesis was that the active r-tACS-induced performance gains would concern, to a greater extent, higher retention loads (i.e., 5 and 7 dots) compared to lower retention loads (1 and 3 dots), in keeping with earlier findings that greater γ-tACS effects can be achieved as the retention load increases in the n-back task [21].

The final experimental prediction concerned the possible maintenance of active r-tACS-induced VSWM performance gains at the two-week follow-up session, as suggested by evidence showing long-term benefits up to a month [29].

## 2. Materials and Methods

### 2.1. Participants

A total of 35 young adult healthy participants (27 women, 5 left-handed, with a mean age of 23.2 ± 4.94 ys) took part in the study. They were recruited among students from psychology master classes in exchange for course credits. In an online screening session, they completed a socio-demographic and medical history questionnaire. The inclusion criteria were normal or corrected-to-normal vision; and having no current or past history of neurologic or psychiatric disorders, a learning disability or the current use of psychoactive medications.

In a randomized single blind protocol, the participants were assigned to one of two groups: active intervention (N = 18) and sham treatment (N = 17). The characteristics of the two groups are shown in Table 2 below. The study included three sessions of active or sham stimulation 24 h from each other at the same time of the day. Each session was composed of three blocks, during which participants performed the behavioral task before stimulation, during stimulation [to test *online* effects], and after stimulation [to test *offline* effects]. Each block lasted 20 min and was separated by a short resting pause (about 1 min). Finally, in order to investigate long-term effects, a follow-up session was conducted after about 2 weeks. A single block of the behavioral task, lasting 20 min, was administered without stimulation. An illustration of the study design is shown in Figure 1.

### 2.2. Experimental Task

Participants sat in a sound-attenuated room facing a computer screen which was at a distance of 60 cm from their eyes. In the spatial capacity delayed response task (SCDRT, [6,7]), each trial started with the presentation of the target display, consisting of an array of 1, 3, 5, or 7 yellow circles positioned pseudorandomly around a central fixation cross for a duration of 0.5 s. After a delay interval of 2.7 to 3.3 s (average 3 s), during which they saw only the fixation cross, the probe display appeared, consisting of a single green circle lasting 0.5 s. Participants were asked to determine if the probe circle was or was not in the same spatial location as one of the preceding target circles by pressing one of two buttons on the computer keyboard (M or Z). Half of the trials contained a probe in the same spatial location (true positives), and half in a different spatial location (true negatives). Each trial ended with an inter-trial interval of 0.8 to 1.3 s, during which the fixation cross was presented alone. A time limit of 1.5 s was set for the choice RT decision. The total duration of each trial was about 5 s.

**Figure 2:**
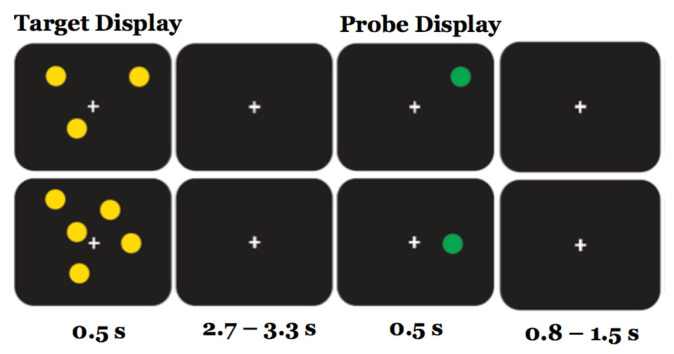
The trial sequence for the SCDRT. Upper row: retention load = 3; lower row: retention load = 5.

Each of the three sessions was preceded by a short practice phase to familiarize the participant with the task, during which visual feedback for a correct or wrong response was provided after each trial. For each session, the three experimental blocks (pre-, during and post-intervention) included 200 trials, divided into two sub-blocks of 100, separated by a short resting pause for a total duration of about 20 min. There were 50 trials for each target capacity load (1, 3, 5 and 7 dots); half were in the same location, while half were different location trials. In each block, the target capacity load (1, 3, 5 and 7) and same/different responses were randomized, with a limit of a maximum of three successive presentations of the same target retention load or the same/different response probe. The RTs and the accuracies of each response were recorded for each target retention time and same/different probe response (Figure 2).

### 2.3. HD-tACS

The alternating current was non-invasively delivered using a five-channel high-definition (HD) transcranial electrical current stimulator (Soterix Medical, Woodbridge, NJ, USA). An elastic cap was embedded with plastic holders containing five 12 mm-diameter Ag/AgCl ring electrodes, filled with conductive gel. The electrodes were placed in a 4 × 1 ring montage, with the central stimulating anode electrode over the left dorsolateral prefrontal cortex (DLPFC), at site F3 of the 10–20 system, corresponding to the MNI coordinates −36, 49, 32 and Brodmann Area 9 [30], and the return cathode electrodes were placed about 5 cm away and approximately equidistant from the neighboring two cathode electrodes (sites F7, Fp1, Fz and C3 of the 10–20 system). A bipolar sinusoidal alternating current was applied at 40 Hz (γ-range) at 1.5 mA intensity for 20 min. All participants tolerated the intervention well, and no adverse events were reported.

**Figure 3:**
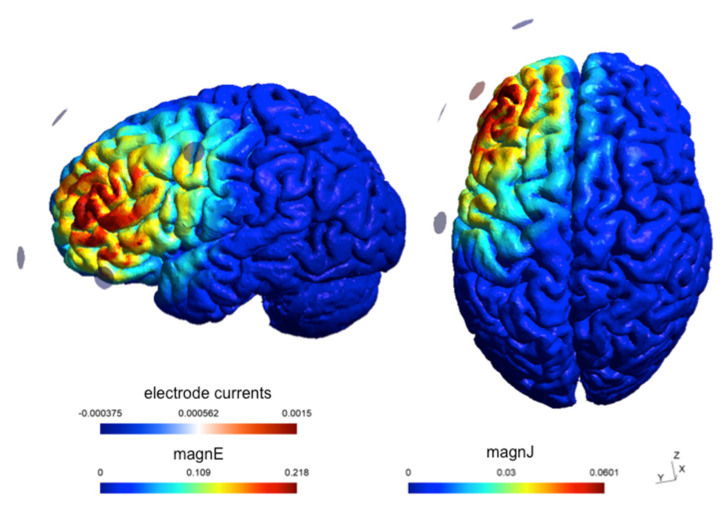
Estimated current flow of the electrical field generated by the γ-tACS at the selected left DLPFC ROI, with the anode placed at F3 and the cathodes placed at F7, Fp1, Fz and C3 according to the international 10–20 system.

The experiment was sham-controlled. The passive sham protocol followed the same procedure as the active neuromodulation procedure, but, critically, the stimulation lasted only 30 s, ramping up and down at the beginning and end of the 20 min period, reproducing the warming and poking sensations participants commonly report and then habituate to during active neuromodulation. Figure 3 depicts the results of a simulation (SimNIBS 4.1 software) modeling the electrical field generated by the γ-tACS and the returning estimates of the electrical field strength (0.18562 V/m) and the current density at the targeted ROI (0.051045 A/m^2^).

### 2.4. Statistical Analysis

Two participants were excluded from further analysis due to technical issues during data collection. Trials with response times faster than 200 ms and slower than 1500 ms were excluded from further analysis. Trials without responses and sub-blocks with less than 70% of responses were excluded (0.018% of experimental trials). We also checked that all participants in the experimental sessions had a mean accuracy of ≥80% in the easiest experimental condition (1 dot). Two participants (one per group) were excluded since they showed <80% mean accuracy in two experimental sessions. The final sample was formed by 33 participants (17 active and 16 sham). Finally, one participant from the active group was excluded from the analysis of the long-term effects of the stimulation since they provided less than 70% of responses in both sub-blocks of the follow-up session.

Two series of statistical analyses were performed, with the independent variables being accuracy and RTs and the dependent variables being Group (active vs. sham stimulation), Session (session 1 to 4), Block (pre-stimulation, stimulation and post-stimulation) and Retention Load (1, 3, 5 or 7), to investigate the following: (a) the short-term effects of active tACS that were analyzed by comparing the performance in the three experimental blocks of sessions one to three; and (b) the long-term effects of active tACS, which included the first experimental block (pre-stimulation) for sessions 1 to 3 as well as the follow-up session. Response accuracy as a dichotomous variable was analyzed using generalized linear mixed models with binomial distribution and logit link. We set nAGQ = 0 to reduce the computational burden. The RTs of correct responses were log-transformed and analyzed using linear mixed-effects models (Gaussian distribution). The mixed-effects models were fitted using the lmer and glmer functions of the lme4 package, respectively [31]. For each dependent variable, model selection was performed using the buildmer function from the “buildmer” package [32]: this function identified the ‘maximal converging model’, which was the model containing either all effects specified by the user, or a subset of those effects that still allowed the model to converge, ordered such that the most information-rich effects were included. Then, it performed a backward stepwise elimination process to select the predictors to be included in the final model. The stepwise elimination was based on the Akaike Information Criterion (AIC) as an index of the goodness of fit of the model. The AIC gave information on the models’ relative evidence (i.e., likelihood) and penalized redundancy; therefore, the model with the lowest AIC was preferred [33,34].

For the analysis of the short-term effects of stimulation, the starting model included the main effects of the dependent variables Session (3 levels: 1st, 2nd and 3rd session), Block (3 levels: 1st, 2nd and 3rd block), WM load (4 levels: 1, 3, 5 and 7 dots) and Group (2 levels: active, sham). The 2-way and 3-way interactions between Session, Block, WM Load and Group were included in the starting model. For the analysis of the long-term effects of the stimulation, the starting model included the main effects of the dependent variables Session (4 levels: 1st, 2nd, 3rd and follow-up session), WM Load and Group. The 2-way interactions and the 3-way interactions between Session, WM Load and Group were included in the starting model. All the tested models included the participants as random intercepts to account for the participant-specific variability and the correlation of the observations within participants. Model outliers were identified using the outlierTest function of the car package [35] and removed (max 1 observation per model). Sum coding was used as contrast coding in order to estimate the main effects [36]. Post hoc comparisons were performed using the contrast function of the emmeans package [37]. The *p*-values were adjusted using Bonferroni correction [38].

## 3. Results

### 3.1. Demographic Variables and Descriptive Statistics

Descriptive statistics for the accuracy according to Group, Session and Block are reported in Table 3.

The descriptive statistics for RTs (msec) according to Group, Session and Block are reported in Table 4.

The descriptive statistics including WM Load are reported in Supplementary Materials (Section S1.1).

#### 3.1.1. Short-Term Effects of Stimulation

##### Accuracy

The best fitting model for response accuracy was Accuracy ~ 1 + WM Load+ Block + Session + WM Load × Block + Block × Session + (1 | Participant).

The test statistics for the best fitting model for response accuracy are reported in Table 5.

The best fitting model for response accuracy did not include the main effect of Group or the related interaction terms.

##### Reaction Times (RTs)

The best fitting model for response times was LogRTs ~ 1 + WM Load + Session + Block + Group + Session × Block + Session × Group + WM Load × Block + WM Load × Session + Block × Group + Session × Block × Group + (1 | Participant).

The test statistics for the best fitting model for response times are reported in Table 6.

### 3.2. Mixed Models Results

Based on our experimental hypotheses, we focused on the interaction effects involving the dependent variable Group, namely Session × Group, Block × Group and Block × Session × Group.

#### 3.2.1. Session × Group Interaction

**Figure 4:**
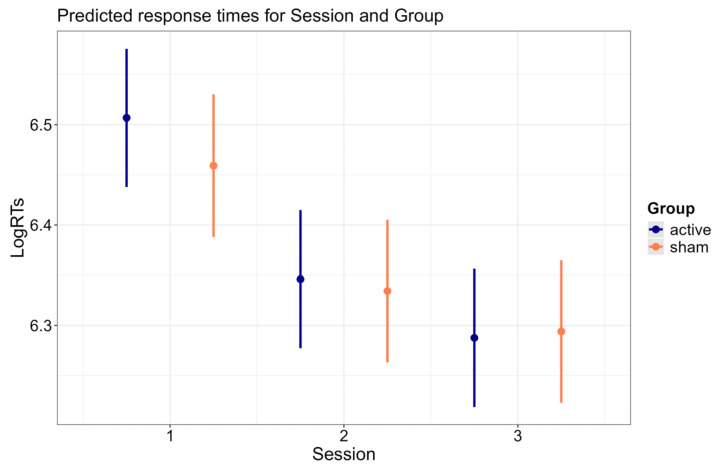
Predicted response times for Session and Group. Error bars indicate 95% confidence intervals.

Figure 4 illustrates the predicted response times for the interaction Session × Group (*p* < 0.001). Both groups displayed faster RTs in the second session compared to the first session (*active*: b = −0.161, SE = 0.004, z-ratio= −41.439, *p* < 0.001; *sham*: b = −0.125, SE = 0.004, z-ratio = −31.254, *p* < 0.001), and in the third session compared to the second session (*active*: b = −0.059, SE = 0.004, z-ratio = −15.295, *p* < 0.001; *sham*: b = −0.041, SE = 0.004, z-ratio = −10.347, *p* < 0.001). The contrasts between the active and sham groups did not attain significance. The Session × Group interaction was explained by a greater reduction in RTs from the first session to the second session (b = 0.036, SE = 0.006, z-ratio = 6.434, *p* < 0.001), as well from the second session to the third session (b = 0.018, SE = 0.006, z-ratio = 3.282, *p* = 0.001) in the active group compared to the sham group.

#### 3.2.2. Block × Group Interaction

**Figure 5:**
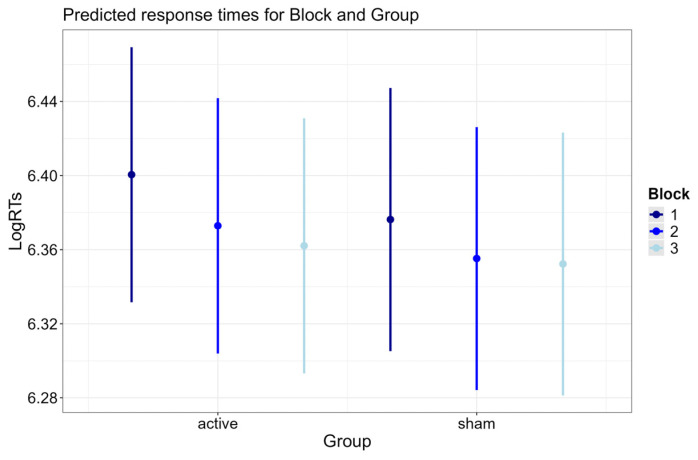
Predicted response times for Block and Group. Error bars indicate 95% confidence intervals.

Figure 5 illustrates the predicted response times for the interaction Block × Group (*p* = 0.035). The significant Block × Group interaction was explained by the fact that, independent of the session, the active group displayed significantly faster RTs in the third block (post-stimulation or *offline* condition) compared to the second block (stimulation or *online* condition) (b = −0.012, SE = 0.004, z-ratio = −2.956, *p* = 0.022), while the sham group showed no differences between the third and second block (b = −0.004, SE = 0.004, z-ratio = −0.876, *p* = 1). No comparison between the active and sham groups reached statistical significance.

#### 3.2.3. Block × Session × Group Interaction

The interaction Block × Session × Group improved model fitting but did not reach statistical significance, albeit it marginally approached significance (*p* = 0.068). Given the evidence that r-tACS improves WM performance over the course of four repetitions [23] and that the effects tACS has on cognitive performance appear to be stronger for *offline* relative to *online* stimulation [19,20], we performed on an exploratory basis, pairwise contrasts in order to explore the specific differences in the estimated marginal means of Block, Session and Group. Such contrasts are reported in the Supplementary Materials (Section S1.2).

### 3.3. Long-Term Effects of Stimulation

#### 3.3.1. Accuracy

The best fitting model for response accuracy was Accuracy ~ 1 + WM Load + Session + (1 | Participant).

The test statistics for the best fitting model are reported in Table 7.

The best fitting model for response accuracy did not include the main effect of Group or the related interaction terms.

#### 3.3.2. Reaction Times (RTs)

The best fitting model for response times was LogRTs ~ 1 + Session + WM Load + Group + Session × Group + Session × WM Load + (1 | Participant).

The test statistics for the best fitting model for response times are reported in Table 8.

Based on our experimental hypotheses, we focused on the interaction effects involving the variable Group, namely Session × Group, Block × Group and Block × Session × Group.

### 3.4. Session × Group Interaction

**Figure 6:**
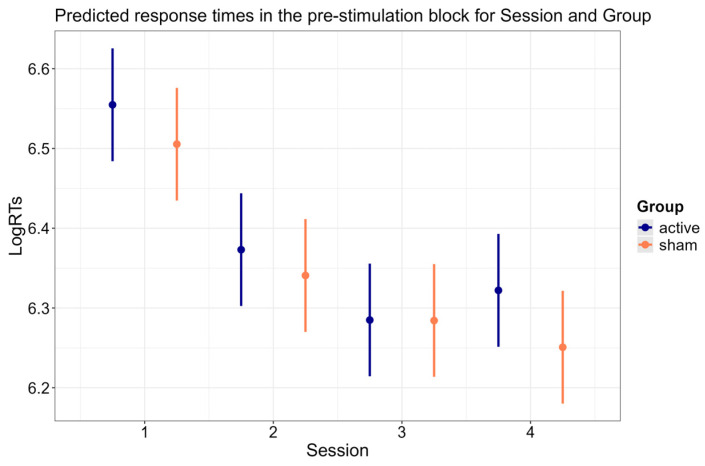
Predicted response times in the pre-stimulation block for Session and Group. Error bars indicate 95% confidence intervals.

Figure 6 illustrates the predicted response times for the interaction between Session and Group (*p* < 0.001).

Both groups showed faster RTs in the pre-stimulation block of the second session compared to the pre-stimulation block of the first session (*active*: b = −0.182, SE = 0.007, z-ratio = −27.312, *p* < 0.001; *sham*: b = −0.165, SE = 0.007, z-ratio = −24.904, *p* < 0.001), and in the pre-stimulation block of the third session compared to the pre-stimulation block of the second session (*active*: b = −0.089, SE = 0.007, z-ratio = −13.484, *p* < 0.001; *sham*: b = −0.057, SE = 0.007, z-ratio = −8.630, *p* < 0.001). The interaction between Session and Group was explained by the greater reduction in RTs in the pre-stimulation block between the second session and the third session in the active group compared to the sham group (b = 0.032, SE = 0.009, z-ratio = 3.405, *p* = 0.004). In contrast, such modulation of the RTs with active stimulation was not present between the first session and the second session (b = 0.017, SE = 0.009, z-ratio = 1.820, *p* = 0.413). Furthermore, and critically, while the sham group showed faster RTs in the follow-up session compared to the pre-stimulation block of the third session (b = −0.034, SE = 0.007, z-ratio = −5.136, *p* < 0.001), the active group showed *increased* response times in the follow-up session compared to the pre-stimulation block of the third session (b = 0.037, SE = 0.007, z-ratio = 5.617, *p* < 0.001). As a further confirmation that the response speed gains due to the active treatment did not propagate to the follow-up session, the active group did not differ from the sham group in the reduction in RTs when comparing the pre-stimulation block of the first session to the follow-up session (b = −0.022, SE = 0.009, z-ratio = −2.330, *p* = 0.119).

## 4. Discussion

This randomized single blind placebo-controlled study was carried out to test the short-term and long-term efficacy of repetitive HD γ-tACS over the left DLPFC in improving the VSWM performance of healthy young adults in the spatial capacity delayed response task (SCDRT). The design allowed us to assess the influence of stimulation type (active vs. passive sham), sessions of stimulation (day 1 to day 3), session block (before stimulation, during stimulation and after stimulation) and VSWM retention load (1 to 7 stimuli).

We present novel evidence for a selective improvement in VSWM performance over the course of three repeated sessions in young adults through the entrainment of gamma rhythms in the left DLPFC in the active stimulation group. The behavioral effects concerned response speed but not accuracy. Such VSWM performance gains in the active stimulation group appeared no longer evident in the long-term at a follow-up session after two weeks.

### 4.1. Effects on RTs and Not Accuracy

We only found that tACS had significant effects for the RT measure. The lack of accuracy effects may be due to a selection bias towards high-performing participants (highly educated healthy young controls) with a mean accuracy of at least 85%. We also excluded participants with less than 80% accuracy and sub-blocks with less than 70% accuracy in order to eliminate outliers that would contaminate the RT analysis. By doing so, we may have reduced our ability to detect accuracy differences.

### 4.2. Short-Term Effects of γ-tACS Repetition (24 h)

Our first experimental hypothesis, that active r-tACS intervention would significantly improve VSWM performance relative to the sham treatment by a progressive boosting of VSWM over the three sessions of stimulation, was upheld. The first mixed model analysis of RTs showed that response speed was significantly greater for the active tACS than the sham group, above and beyond the effects of practice or other effects due to expectation, with significant gains in sessions 2 and 3. This effect was present regardless of the experimental block (before, during or after stimulation).

The second mixed model analysis, restricted to the baseline pre-stimulation condition (block 1) of each session, confirmed the greater gains in RTs over consecutive sessions for the active intervention. The effect was, in this case, significant for session 3, while it did not approach significance for session 2. Combining the results from both analyses, it appears safe to conclude that active HD γ-tACS produced significant and incremental VSWM gains lasting at least until the following stimulation session (24 h), starting from session 2.

To our knowledge, only one recent study has explored the effects of r-tACS (four sessions) on WM performance, reporting WM benefits in session 3 and session 4. However, the task involved audio verbal WM, the stimulation locus was over the parietal region and the effective stimulation frequency was in the θ-range (4–7 Hz) and not in the γ-range. Furthermore, the study involved an aging population (65–88 ys old) [29].

In the literature, repeated sessions of α (8–12 Hz)-HD-tACS also yielded significant effects in other domains, such as in the study of the effects of anxiety. Four sessions of α-HD-tACS over the parieto-occipital scalp were effective in reducing anxiety symptoms and related EEG functional connectivity correlates both at 30 min and after 24 h [39].

This was also consistent with previous research suggesting that increasing the number of tACS sessions can significantly improve responses to stimulation, leading to cumulative and long-lasting effects through mechanisms that promote neuroplasticity, which is particularly beneficial for older adults or clinical populations [27,28].

It has been proposed that the WM benefits of tDCS of the left DLPFC in an n-back task may be explained by a mechanism of long-term potentiation (LTP): a short episode of synaptic activation in the stimulated area inducing a persistent increase in synaptic transmission through neuronal plasticity due to the synthesis of genic products [16]. The same mechanism of brain plasticity has been advocated to explain the long-term effects of repetitive and highly focused tACS targeting memory-specific cortical regions and the proper oscillatory band [27,28,29].

In conclusion, this is the first study demonstrating significant VSWM gains by employing the SCDRT with incremental effects over three days of γ-HD-tACS over the left DLPFC in healthy young adults, and which corroborates the efficacy of repeated sessions of stimulation to boost cognitive performance, contrary to several studies in the literature reporting no effects of single sessions of tACS on WM performance in healthy populations [22,23,24]. This study provides further rationale for employing multiple sessions of stimulation to produce more robust gains in cognitive performance [29,39], with important implications for the utility of r-tACS to improve cognitive performance in aging and clinical populations with various neuropsychiatric conditions.

### 4.3. Short-Term Effects of Online vs. Offline γ-tACS (30 min)

Our second experimental hypothesis that active r-tACS would benefit VSWM performance significantly more in the post-stimulation block (*offline* effect) rather than during stimulation (*online* effect) was also supported. Across the three days of intervention, active γ-tACS relative to the sham tACS improved RT performance significantly in the post-stimulation block (block 3) relative to the stimulation block (block 2). This is in line with the results of previous meta-analyses employing both tDCS and tACS in healthy cohorts, which reported similar advantages of *offline* over *online* stimulation in improving WM performance. The present study extends this finding to also include VSWM assessed by the SCDRT. The greater offline effects of tACS could also explain the limited effects in several single session studies in which tACS was only applied online—during the execution of the task [22,25,26].

The *offline* stimulation effect can be more easily accounted for by the causal mechanism of tACS-induced neural entrainment promoting the synchronization of phasic neural oscillations at specific frequencies, if we assume that it would take some time to build up the effect, which would then manifest at a greater extent in the *offline* period (30 min from stimulation) than the *online* period. This mechanism could explain the similar short-lived gains of tACS (30 min) reported in the literature [39], rather than it being caused by LTP and brain plasticity.

### 4.4. Short-Term Effects of WM Load

Our third hypothesis that active r-tACS-induced VSWM performance gains would concern, to a greater extent, higher retention loads (i.e., 5 and 7 dots) compared to lower retention loads (1 and 3 dots) was not upheld. There was no hint that response speeds or accuracy in the SCDRT were significantly affected by VSWM retention load. This result contradicts the findings of a study reporting greater gains in WM in an n-back task with γ-tACS of the left DLPFC, as the retention load increased in healthy participants from 2-back to 3-back [21], and contradicts similar claims that combined γ-tACS and θ-tACS stimulation of the left frontal cortex improved the WM performance of young adults in the Sternberg task for the high load condition [25]. We can only speculate that differences in task difficulty or performance level may explain at least in part such a discrepancy. In the present study, accuracy was very high (>85% on average), and there is evidence that the effects of neurostimulation may be more pronounced in participants with lower compared to higher cognitive performance [29]. It is also worth mentioning that a statistically robust effect of tACS on cognitive load in WM tasks may be elusive, because it would require a considerably larger sample size, with the significance of a two- or even three-way interaction (i.e., Group × Stimulation type × Retention Load). In fact, in the study reporting γ-tACS effects on cognitive load in the n-back task, the Stimulation × Load interaction was not significant (*p* = 0.09), and the tACS effect on 3-back relative to 2-back was explained by an a priori hypothesis [21].

### 4.5. Long-Term Effects of γ-tACS

Our final experimental hypothesis that active r-tACS-induced VSWM performance gains would persist long-term, at the two-week follow-up session, was also not supported. The benefits of γ-tACS on VSWM response speed over the sham treatment which were present at sessions 2 and 3 were no longer evident in the follow-up session, after two weeks. This result seems to be in contrast with the effectiveness of long-term stimulation (up to a month) reported in another WM study employing four repeated sessions in elderly healthy participants [29]. It is possible that a long-term VSWM effect would need additional stimulation sessions to be appreciable, at least in young healthy cohorts, providing some rationale for using an increased number of repeated sessions to achieve cognitive or clinical benefits.

### 4.6. Caveats and Future Directions

Our study was only carried out at a behavioral level—the combination of the recordings of brain electrical activity before, during and after tACS stimulation may have revealed important effects on brain function [24], as well as allowing us to adjust the stimulation frequency to the exact γ-band peak for each individual. Behavioral WM effects may have been more pronounced by stimulating simultaneously the left frontal and parietal nodes of the known VSWM fronto-parietal network [6,7], as recent tACS studies have shown [24].

Furthermore, our study included two relatively small samples of healthy young participants drawn from a rather homogeneous well-educated university student community, with very high-performance accuracy (>85%), so that accuracy effects due to participants with lower cognitive performance could not be detected, as well as the possible effects of r-tACS on older people [29].

Future studies, with a larger sample size, may evaluate the influence of age, gender and dispositional factors, such as traits of anxiety or depression.

## 5. Conclusions

All of the above limitations notwithstanding, this is to our knowledge the first study to report the VSWM benefits of repetitive HD γ-tACS of the left DLPFC in healthy young adults, finding the short-term effects of repetition over three repeated sessions as well as the effects of offline vs. online stimulation. More studies in the future with additional stimulation sessions could yield more consistent long-term cognitive performance or clinical benefits. Importantly, the potential for clinical efficacy could be validated in patients with neuropsychiatric disorders with various levels of WM dysfunction, such as clinical depression, anxiety or mild cognitive impairment.

## Figures and Tables

**Table 2:** Demographic variables (mean ± sd) per group (active, sham); N = number of participants; % = percent of participants; ns: not significant. *: unpaired *t*-test; ^$^: Chi-square independence test.

Participant Variables	Active	Sham	Comparison
N	18	17	
Age (years)	21.61 ± 1.29	24.71 ± 6.72	ns *
Education (years)	16.06 ± 1.26	17 ± 2.45	ns *
Male (N, %)	2 (11%)	5 (29%)	ns ^$^
Right-Handed (N, %)	17 (94%)	13 (76%)	ns ^$^

**Table 3:** Mean accuracy and standard deviations for Group, Session and Block.

	Active			Sham		
	Block 1	Block 2	Block 3	Block 1	Block 2	Block 3
Session 1	0.85 ± 0.05	0.84 ± 0.06	0.84 ± 0.08	0.85 ± 0.07	0.85 ± 0.08	0.86 ± 0.09
Session 2	0.85 ± 0.08	0.84 ± 0.07	0.85 ± 0.07	0.85 ± 0.09	0.86 ± 0.08	0.85 ± 0.10
Session 3	0.87 ± 0.07	0.85 ± 0.08	0.84 ± 0.08	0.87 ± 0.08	0.85 ± 0.07	0.86 ± 0.09
Follow-up	0.86 ± 0.07			0.86 ± 0.09		

**Table 4:** Mean response times and standard deviations for Group, Session and Block.

	Active			Sham		
	Block 1	Block 2	Block 3	Block 1	Block 2	Block 3
Session 1	726 ± 212	690 ± 209	663 ± 200	691 ± 217	653 ± 215	647 ± 210
Session 2	607 ± 190	590 ± 191	585 ± 190	589 ± 193	586 ± 190	581 ± 188
Session 3	558 ± 176	559 ± 189	568 ± 194	559 ± 177	564 ± 188	569 ± 184
Follow-up	580 ± 182			540 ± 171		

**Table 5:** Test statistics for the best fitting model for response accuracy.

	Chisq	Df	*p*-Value
Intercept	430.545	1	<0.001
WM Load	1147.786	3	<0.001
Block	18.983	2	<0.001
Session	9.152	2	0.010
WM Load × Block	29.120	6	<0.001
Block × Session	8.021	4	0.091

**Table 6:** Test statistics for the best fitting model for response times.

	Chisq	Df	*p*-Value
Intercept	64,099.766	1	<0.001
WM Load	6237.970	3	<0.001
Session	5154.541	2	<0.001
Block	155.666	2	<0.001
Group	0.122	1	0.727
Session × Block	225.740	4	<0.001
**Session × Group**	97.406	2	**<0.001**
WM Load × Block	56.954	6	<0.001
WM Load × Session	53.370	6	<0.001
**Block × Group**	6.681	2	**0.035**
Block × Session × Group	8.741	4	0.068

**Table 7:** Test statistics for the best fitting model for response accuracy.

	Chisq	Df	*p*-Value
Intercept	465.179	1	<0.001
WM Load	513.608	3	<0.001
Session	23.283	3	<0.001

**Table 8:** Test statistics for the best fitting model for response times.

	Chisq	Df	*p*-Value
Intercept	63,235.497	1	<0.001
Session	3670.141	3	<0.001
WM Load	3383.867	3	<0.001
Group	0.576	1	0.448
**Session × Group**	61.837	3	**<0.001**
Session × WM Load	30.470	9	<0.001

## Data Availability

Data presented in this study are stored and kept in an archived form by the supervisor of the study (M.L.). Data may be available on request from the corresponding author due to privacy reasons.

## Supplementary Materials

The following supporting information can be downloaded at: https://www.mdpi.com/article/10.3390/brainsci15040343/s1.
