# Supplementary material for: Repetitive Gamma-tACS Improves the Reaction Times of Healthy Young Adults in a Visuospatial Working Memory Task: A Randomized Study
Source: Brain Sci. 2025 Mar 27;15(4):343. doi: 10.3390/brainsci15040343 (PMC12026391; doi:10.3390/brainsci15040343)
Supplement: Supplementary file 1 [file brainsci-15-00343-s001.zip › brainsci-3506183-supplementary.pdf]

Supplementary Materials

S1.1. Descriptive Statistics Including WM Load

The descriptive statistics for mean accuracy according to Group, Session, Block and WM Load are reported in Tables S1 and S2 below.

Table S1. Mean accuracy and standard deviations for Group, Session, WM Load and Block.

		Active			Sham		
		**Block 1**	**Block 2**	**Block 3**	**Block 1**	**Block 2**	**Block 3**
**Session 1**	**1 Stim**	0.95 ± 0.05	0.94 ± 0.06	0.93 ± 0.07	0.95 ± 0.04	0.94 ± 0.06	0.94 ± 0.05
	**3 Stim**	0.84 ± 0.07	0.81 ± 0.08	0.80 ± 0.13	0.83 ± 0.10	0.81 ± 0.09	0.83 ± 0.11
	**5 Stim**	0.80 ± 0.09	0.81 ± 0.08	0.84 ± 0.10	0.82 ± 0.09	0.81 ± 0.10	0.85 ± 0.10
	**7 Stim**	0.79 ± 0.07	0.79 ± 0.11	0.78 ± 0.08	0.81 ± 0.10	0.82 ± 0.12	0.80 ± 0.12
**Session 2**	**1 Stim**	0.95 ± 0.05	0.93 ± 0.05	0.91 ± 0.07	0.95 ± 0.04	0.94 ± 0.05	0.93 ± 0.07
	**3 Stim**	0.83 ± 0.10	0.79 ± 0.09	0.82 ± 0.09	0.84 ± 0.11	0.83 ± 0.13	0.82 ± 0.13
	**5 Stim**	0.82 ± 0.10	0.81 ± 0.09	0.84 ± 0.09	0.83 ± 0.12	0.82 ± 0.09	0.83 ± 0.12
	**7 Stim**	0.80 ± 0.10	0.82 ± 0.09	0.81 ± 0.08	0.78 ± 0.12	0.83 ± 0.09	0.80 ± 0.13
**Session 3**	**1 Stim**	0.96 ± 0.04	0.94 ± 0.08	0.91 ± 0.08	0.94 ± 0.05	0.93 ± 0.06	0.94 ± 0.07
	**3 Stim**	0.84 ± 0.11	0.80 ± 0.10	0.81 ± 0.10	0.85 ± 0.10	0.84 ± 0.08	0.84 ± 0.10
	**5 Stim**	0.85 ± 0.10	0.84 ± 0.09	0.82 ± 0.08	0.86 ± 0.09	0.83 ± 0.10	0.85 ± 0.10
	**7 Stim**	0.82 ± 0.08	0.81 ± 0.08	0.81 ± 0.09	0.84 ± 0.10	0.80 ± 0.12	0.82 ± 0.12
**Follow-up**	**1 Stim**	0.95 ± 0.05			0.93 ± 0.06		
	**3 Stim**	0.84 ± 0.10			0.85 ± 0.11		
	**5 Stim**	0.85 ± 0.09			0.85 ± 0.12		
	**7 Stim**	0.82 ± 0.09			0.82 ± 0.10		

Table S2. Mean response times and standard deviations for Group, Session, WM Load and Block.

		Active			Sham		
		**Block 1**	**Block 2**	**Block 3**	**Block 1**	**Block 2**	**Block 3**
**Session 1**	**1 Stim**	609 ± 177	590 ± 173	577 ± 172	586 ± 186	565 ± 197	572 ± 204
	**3 Stim**	759 ± 210	718 ± 195	691 ± 195	725 ± 210	687 ± 211	682 ± 198
	**5 Stim**	779 ± 205	744 ± 220	700 ± 207	729 ± 215	683 ± 205	673 ± 207
	**7 Stim**	782 ± 204	727 ± 209	700 ± 200	744 ± 219	690 ± 221	671 ± 209
**Session 2**	**1 Stim**	515 ± 158	507 ± 163	506 ± 164	502 ± 159	510 ± 170	519 ± 173
	**3 Stim**	631 ± 177	610 ± 187	613 ± 181	617 ± 200	610 ± 178	597 ± 179
	**5 Stim**	643 ± 192	629 ± 192	606 ± 189	627 ± 199	622 ± 198	601 ± 185
	**7 Stim**	655 ± 197	625 ± 195	623 ± 201	623 ± 184	614 ± 198	619 ± 200
**Session 3**	**1 Stim**	483 ± 143	496 ± 160	503 ± 168	486 ± 152	502 ± 186	505 ± 169
	**3 Stim**	581 ± 179	569 ± 170	594 ± 198	570 ± 162	581 ± 181	588 ± 179
	**5 Stim**	578 ± 170	587 ± 199	591 ± 204	589 ± 187	591 ± 183	596 ± 187
	**7 Stim**	601 ± 189	595 ± 206	593 ± 190	599 ± 184	591 ± 186	595 ± 186
**Follow-up**	**1 Stim**	511 ± 169			465 ± 152		
	**3 Stim**	598 ± 178			565 ± 166		
	**5 Stim**	599 ± 180			569 ± 166		
	**7 Stim**	619 ± 183			571 ± 174		

S1.2. Mixed Model Analysis: Block × Session × Group interaction

Figure S1 shows the estimated marginal means for Block, Session and Group.

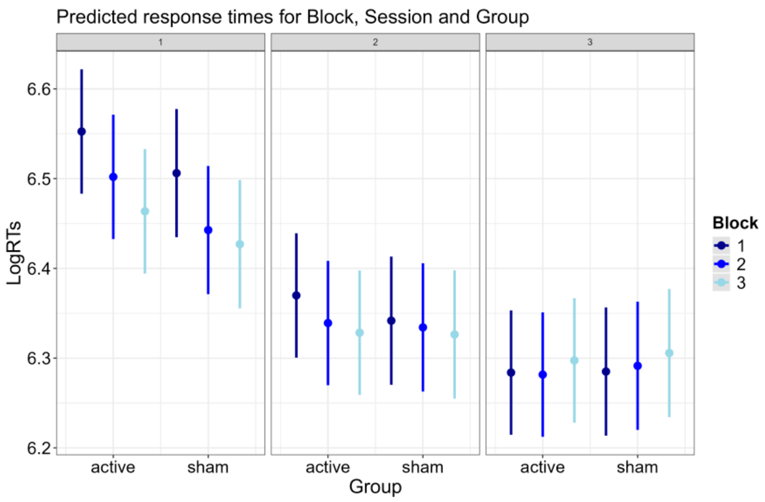

Figure S1. Predicted response times for Block, Session and Group. Error bars indicate 95% confidence intervals.

Results of the exploratory analysis on the Block × Session × Group interaction ($p < 0.068$): In the first tACS session, both groups showed faster RTs in the second block (*online* stimulation) compared to the first baseline block (*active*: b = −0.051, SE = 0.007, z-ratio = −7.650, $p < 0.001$; *sham*: b = −0.064, SE = 0.007, z-ratio = −9.395, $p < 0.001$). The active tACS group showed faster RTs in the third block (*offline* stimulation) relative to the second block (*online* stimulation; b = −0.039, SE = 0.007, z-ratio = −5.725, $p < 0.001$), while the sham group showed no differences between the third and second block (b = −0.016, SE = 0.007, z-ratio = −2.282, $p = 0.562$). In the second tACS session, the active group showed faster RTs in the second block (*online* stimulation) compared to the first baseline block (b = −0.032, SE = 0.007, z-ratio = −4.725, $p < 0.001$), while the sham group showed no differences (b = −0.008, SE = 0.007, z-ratio = −1.203, $p = 1$). Both groups showed no differences between the third block (*offline* stimulation) and the second block (online stimulation; *active*: b = −0.011, SE = 0.007, z-ratio = −1.662, $p = 1$; *sham*: b = −0.008, SE = 0.007, z-ratio = −1.208, $p = 1$). In the third tACS session, both groups showed no differences between the second and the first block (*active*: b = −0.003, SE = 0.007, z-ratio = −0.457, $p = 1$; *sham*: b = 0.006, SE = 0.007, z-ratio = 0.830, $p = 1$) and between the third and second block (*active*: b = 0.015, SE = 0.007, z-ratio = 2.299, $p = 0.538$; *sham*: b = 0.014, SE = 0.007, z-ratio = 2.041, $p = 1$). No comparison between the sham and active groups reached statistical significance.
